# Quantitative macromolecular patterns in phytoplankton communities resolved at the taxonomical level by single-cell Synchrotron FTIR-spectroscopy

**DOI:** 10.1186/s12870-019-1736-8

**Published:** 2019-04-15

**Authors:** Andrea Fanesi, Heiko Wagner, Giovanni Birarda, Lisa Vaccari, Christian Wilhelm

**Affiliations:** 1 Department of Plant Physiology, Leipzig University, Institute of Biology, Johannisallee 21-23, 04103 Leipzig, Germany; 2 German Centre for Integrative Biodiversity Research (iDiv) Halle-Jena-Leipzig, Deutscher Platz 5e, 04103 Leipzig, Germany; 3 Elettra - Sincrotrone Trieste, Synchrotron Infrared Source for Spectroscopy and Imaging – SISSI, 34149 Trieste, Basovizza Italy

**Keywords:** Algae, Chemometrics, FTIR-spectroscopy, Macromolecules, Phytoplankton, PLSr, Single cell, Synchrotron radiation, Temperature

## Abstract

**Background:**

Technical limitations regarding bulk analysis of phytoplankton biomass limit our comprehension of carbon fluxes in natural populations and, therefore, of carbon, nutrients and energy cycling in aquatic ecosystems. In this study, we took advantage of Synchrotron FTIR micro-spectroscopy and the partial least square regression (PLSr) algorithm to simultaneously quantify the protein, lipid and carbohydrate content at the single-cell level in a mock phytoplankton community (composed by a cyanobacterium, a green-alga and a diatom) grown at two temperatures (15 °C and 25 °C).

**Results:**

The PLSr models generated to quantify cell macromolecules presented high quality fit (R^2^ ≥ 0.90) and low error of prediction (RMSEP 2–6% of dry weight). The regression coefficients revealed that the prediction of each macromolecule was not exclusively dependent on spectral features corresponding to that compound, but rather on all major macromolecular pools, reflecting adjustments in the overall cell carbon balance.

The single-cell analysis, studied by means of Kernel density estimators, showed that the modes of density distribution of macromolecules were different at 15 °C and 25 °C. However, a substantial proportion of cells was biochemically identical at the two temperatures because of population heterogeneity.

**Conclusions:**

The spectroscopic approach presented in this study allows the quantification of macromolecules in single phytoplankton cells. This method showed that population heterogeneity most likely ensures a backup of non-acclimated cells that may rapidly exploit new favourable niches. This finding may have important consequences for the ecology of phytoplankton populations and shows that the “average cell” concept might substantially limit our comprehension of population dynamics and biogeochemical cycles in aquatic ecosystems.

**Electronic supplementary material:**

The online version of this article (10.1186/s12870-019-1736-8) contains supplementary material, which is available to authorized users.

## Background

The assimilation of inorganic nutrients into organic macromolecules allows phytoplankton growth. The genotype and the environment define the macromolecular stoichiometry (i.e. proteins:lipids:carbohydrates) of new cells and therefore the electrons and carbon (C) required to produce them [1]. Thus, a precise quantification of cell macromolecules would shed light on the cost of biomass production and on its quality [2–4]. From a physiological point of view, this may reveal interesting acclimation pattern at different growth conditions, whereas from an ecological perspective it could provide important information concerning the energy transfer in food-webs and about biogeochemical cycles in aquatic ecosystems [5–7].

Although the identification of common acclimation strategies to environmental changes may help to model primary production in nature [2], recent experimental studies have reported that the adjustments in C-allocation pattern are strongly species-specific in response to a determinate external perturbation [4, 7, 8]. It is also interesting to notice that within a clonal phytoplankton population relative macromolecular differences among cells have been observed [9, 10], due to a drastic population heterogeneity in gene expression [11]. This in turn limits our comprehension of phytoplankton dynamics in nature, as well as our ability to model primary production. Predictive models are indeed not considering such a high resolution of adjustments. However, recent ecological theories have been scaled down at the single-cell level (virus-phytoplankton, bacteria-phytoplankton, phytoplankton-phytoplankton interactions etc.), thus, information about the single biological units of a population may be of great help to better understand community dynamics [12]. This high resolution is especially required when considering the great diversity of C-allocation strategies adopted by different taxa (and even species) in response to environmental changes [4, 7, 13, 14].

Up to date, the most used techniques to determine the macromolecular composition of phytoplankton are represented by standard biochemical assays [15] or incubation of samples with C^14^ [16]. However, bulk measurements only deliver the average biochemical profile of a population and neglect the phenotypic variability among single cells. Furthermore, biochemical assays are time consuming and relatively expensive [17, 18]. Other methods use statistics to correlate macromolecules to more accessible cellular traits (i.e. cell characteristics that may describe their growth rate or fitness in a specific environment). Recently, Finkel et al. [6] compiled an updated estimate of the macromolecular composition of phytoplankton and developed predictive linear models for the quantification of macromolecules based on their cellular volume [6]. This study provides an interesting bridge between an easy-measurable trait (i.e. cell volume) and the more complex macromolecular components of phytoplankton biomass.

Approaches such as flow cytometry as well as genomic and transcriptomic analysis may have the potential to resolve phytoplankton traits at the single-cell level [19, 20]. However none of these approaches can provide an overview of macromolecular composition in single phytoplankton cells. On the other hand, Fourier Transform Infrared (FTIR) spectroscopy has been extensively used for the macromolecular characterization of phytoplankton in uni-algal cultures [17, 21, 22]. This spectroscopic technique allows to acquire a rapid and precise snapshot of the total biochemical composition of phytoplankton samples, and presents many advantages with respect to standard chemical bulk analysis [18, 23–25]. It is cheap, fast and can be performed at the single-cell level, when using Synchrotron radiation as IR source in combination with a microscope (Synchrotron FTIR micro-spectroscopy) [7, 10, 14, 26].

The quantification of macromolecules in bulk phytoplankton samples by means of FTIR-spectroscopy has been tested using several approaches [18]. However, an absolute quantification in single-cells is missing so far, indeed to the best of our knowledge the studies present in the literature only reported relative changes in band intensities of single cells [9, 10, 26, 27]. For this purpose, particularly useful is the possibility of coupling FTIR-spectroscopy to advanced statistical analysis (i.e. chemometrics) for the construction of predictive models [8, 23, 28, 29]. Chemometrics can be used to link FTIR-spectra to a reference measurement, creating multivariate calibrations aimed at predicting parameters of unknown samples. In the field of applied phycology, such models provide a fast and reliable method for the estimation of specific cellular traits that would otherwise be difficult to assess [23, 28, 29]. Several cellular traits ranging from elemental ratios to more complex metabolic processes (e.g. growth rate and C production), have already been modeled on the base of FTIR-spectra [8, 23, 28, 29]. However, the applicability of such predictive models has never been tested at the single-cell level.

In this study, we developed an approach that combines both Synchrotron FTIR micro-spectroscopy and chemometric predictive models based on the partial least square regression algorithm (PLSr) [30, 31], to quantify the whole macromolecular composition of single phytoplankton cells. Since natural samples of aquatic communities may contain high amount of organic matter, bacteria and other contaminants, as a first trial to test the application of the method, laboratory cultures were preferred compared to natural samples. In order to mimic the conditions of natural communities, we used defined mixtures of three different phytoplankton species (a green alga, a cyanobacterium and a diatom) to establish mock communities that were subjected to two temperatures. Temperature was chosen as an external factor to induce C-allocation changes because, in the context of global warming, it will play a crucial role affecting phytoplankton productivity and water quality. Nevertheless, the lack of information regarding the effects of temperature on C-allocation among the pool of macromolecules limits the precision of future predictions [25, 32, 33]. Fanesi et al. [28] recently reported temperature-related biochemical alterations of phytoplankton composition on a bulk basis. However, considering the higher resolution allowed by single-cell spectroscopy, the present approach aims at revealing interesting population and community patterns that would otherwise remain unknown. For instance, non-uniform gene expression and random level of transcripts is typically translated in a variety of physiological states and functions that cells may accomplish in a population [34]. We, therefore, hypothesized that due to phenotypic heterogeneity, the response of a population to temperature is not uniform but characterized by different sub-responses arising from the diversity of phenotypes.

Therefore, the aim of this study is to use FTIR spectroscopy to quantify, for the first time, the absolute content of the main macromolecules in single phytoplankton cells, and to determine sub-responses within a plankton community.

## Results

### Macromolecule quantification by reference biochemical methods

The dry weight (DW) of *A. obliquus* and *M. aeruginosa* was not influenced by temperature. On the other hand, temperature appears to affect more the DW of *A. granulata* cells, which was significantly higher at 25 °C (Table 1).

**Table 1:** Dry weight (pg cell^− 1^) and macromolecule contents (% cell dry weight), estimated by biochemical assays, of pure cultures of *M. aeruginosa*, *A. oliquus* and *A. granulata* grown at 15 and 25 °C. Values in parentheses are the standard deviations (*n* = 5). Different letters represent statistically different means (*P < 0.05*). Equal letters represent no significantly different means (*P > 0.05*)

	*M. aeruginosa*		*A. obliquus*		*A. granulata*	
	15 °C	25 °C	15 °C	25 °C	15 °C	25 °C
Dry weight (pg cell^−1^)	13.2^a^ (4.3)	9.6^a^ (0.7)	29.1^a^ (7.6)	21.2^a^ (1.2)	137.9^a^ (25.9)	192.6^b^ (28.2)
Proteins (% DW)	26.9^a^ (±9.9)	44.6^b^ (±11.6)	46.4^a^ (±13.8)	36.7^a^ (±6.4)	16.5^a^ (±8)	21.7^a^ (±6.2)
Lipids (% DW)	38.4^a^ (±9.1)	44.7^a^ (±12.7)	38.8^a^ (±16.9)	46.6^a^ (±9.3)	33.7^a^ (±14.8)	35.4^a^ (±6.7)
Carbohydrates (% DW)	34.6^a^ (±19.7)	10.6^b^ (±1.8)	14.7^a^ (±3.7)	16.6^a^ (±2.8)	5.8^a^ (±0.2)	4^b^ (±1.4)
Silica (% DW)	/	/	/	/	43.8^a^ (±11.4)	38.8^a^ (±8.8)

Irrespective of temperature changes, *A. obliquus* and *M. aeruginosa* showed similar macromolecular compositions (Table 1). Almost 15–35% of the biomass was represented by carbohydrates, 27–46% of DW was constituted by proteins and the remaining fraction was made up by lipids (38–46% of DW). In *A. granulata* almost 40% of DW was represented by Si. The organic fraction of the diatom biomass presented similar amounts of lipids (~ 40% of DW) compared to *A. obliquus* and *M. aeruginosa* but less proteins (16–21% of DW) and carbohydrates (~ 5% of DW) (Table 1).

Temperature induced relevant changes in the macromolecular composition of *A. granulata* and *M. aeruginosa* but not in *A. obliquus* (Table 1). Carbohydrates were significantly higher at 15 °C, with respect to 25 °C, in *A. granulata* and *M. aeruginosa*. Proteins were significantly higher in *M. aeruginosa* at 25 °C with respect to 15 °C. In all the species, the lipid pool was not affected by temperature, the same was true for the silica content of *A. granulata* that resulted unchanged at the two temperatures (Table 1).

### Chemometric analysis of FTIR-spectra using PLSr

**Fig. 1:**
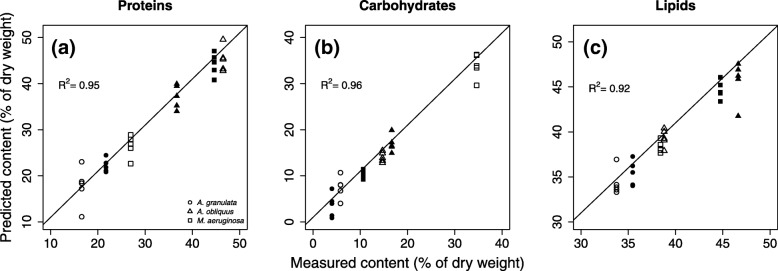
Calibration plot obtained from the PLSr models developed for the determination of proteins (**a**), carbohydrates (**b**) and lipids (**c**) calibrated using bench-top FTIR-spectra and “wet” biochemical assays as a reference method. Open symbols correspond to 15 °C, closed symbols to 25 °C. Each model was calibrated using three phytoplankton species (a diatom [circle], a cyanobacterium [square] and a green alga [triangle]) grown at two temperatures (15 °C and 25 °C). The macromolecule content is expressed as % of dry weight and the R^2^ for each regression is reported. The number of PLSr-PCs used was 7

The calibration plots (Fig. 1) showed that the bench-top FTIR-spectra of uni-algal cultures were highly correlated to the protein, lipid and carbohydrate content of the cells. Using 7 components the R^2^ of the models were 0.94 for proteins, 0.96 for carbohydrates and 0.91 for lipids (Table 2, Fig. 1) and the RMSEC was lower than 3% of DW for all models. The prediction performance of each model was assessed by the leave-one-out cross-validation. The estimated error in predicting unknown samples (RMSEP) was 6.27 (% of DW) for proteins, 2.49 for carbohydrates and 4.89 for lipids (Table 2). The R^2^ of predicted estimates was 0.68 for proteins, 0.76 for carbohydrates and 0.70 for lipids (Additional file 1: Figure S1).

**Table 2:** PLSr summary statistics. R^2^; coefficient of determination obtained from the training set; RMSEC: root mean squared error of calibration obtained from the training set; RMSEP: root mean squared error of prediction obtained after LOOV

	n° of species	Temperature (°C)	n° of calibration spectra	R^2^	RMSEC (% of DW)	RMSEP (% of DW)	n° of factors	% variance explained	Most relevant spectral predictors
Proteins	3	15 & 25	30	0.95	2.6	6.27	7	94.65	CH_2_ and C=O (lipids); Amide I and II (proteins); C-O-C (carbohydrates)
Carbohydrates	3	15 & 25	30	0.96	2	4.84	7	96.03	C-O-C (carbohydrates)
Lipids	3	15 & 25	30	0.92	1.34	2.49	7	91.54	CH_2_ (lipids); Amide I (proteins); C-O-C (carbohydrates)

For each macromolecular pool, the first 7 PLSr principal components (PC) explained most (> 90%) of the variance both related to the descriptor matrix, i.e. the spectral dataset and to the response variables (i.e. proteins, lipids or carbohydrates) (Table 2, Additional file 2: Table S2, Additional file 3: Table S3 and Additional file 4: Table S4).

**Fig. 2:**
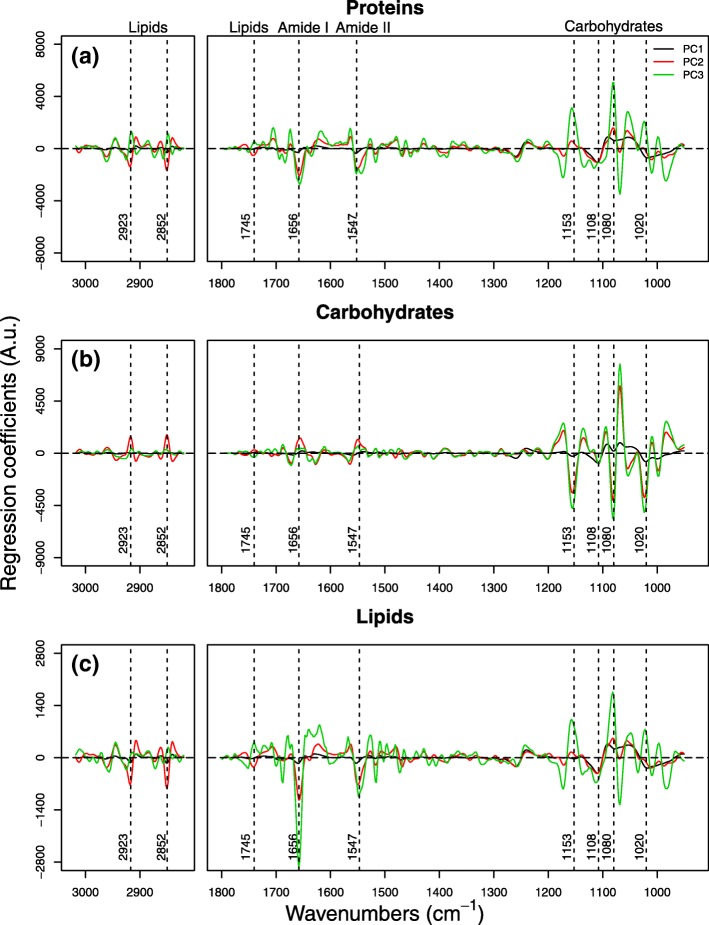
Regression coefficients for the first three PC components of the PLSr models calibrated to predict protein (**a**), carbohydrate (**b**) and lipid (**c**) content in phytoplankton cells. The wavenumbers of the most important coefficients are reported together with the macromolecular assignment of the corresponding bands

The regression coefficients highlight which spectral bands are mostly correlated with the macromolecular changes of the cell composition, and therefore which are the most important peaks for the modeling process. We focused our analysis on the first three PLSr-PCs because explaining the highest percentage of variance in the descriptive and response matrix. The prediction of the three macromolecules was mostly driven by the same spectral features (Fig. 2, Table 2): asymmetric and symmetric CH_2_ stretching modes (2915 and 2848 cm^− 1^), the band related to the stretching of the C=O bond of lipids (at 1740 cm^− 1^), the Amide I (1658 cm^− 1^) and the Amide II (1544 cm^− 1^) bands of proteins. At lower wavenumber, peaks corresponding to carbohydrates (1153, 1108, 1080 and 1020 cm^− 1^) were also identified as important for the models.

The contribution of the spectral features for the prediction of the content of each macromolecule was specific. Protein prediction was driven by lipids (CH_2_ and C=O stretching modes), proteins (Amide I and II) and carbohydrates bands (C-O, and C-O-C). Carbohydrates prediction was based mostly on C-O, and C-O-C bands typical of polysaccharides. Finally, the lipid content estimation was dependent on lipid CH_2_ stretching, Amide I and C-O, C-O-C bands of polysaccharides (Fig. 1, Table 2).

### FTIR 2nd derivative spectra and principal component analysis (PCA)

**Fig. 3:**
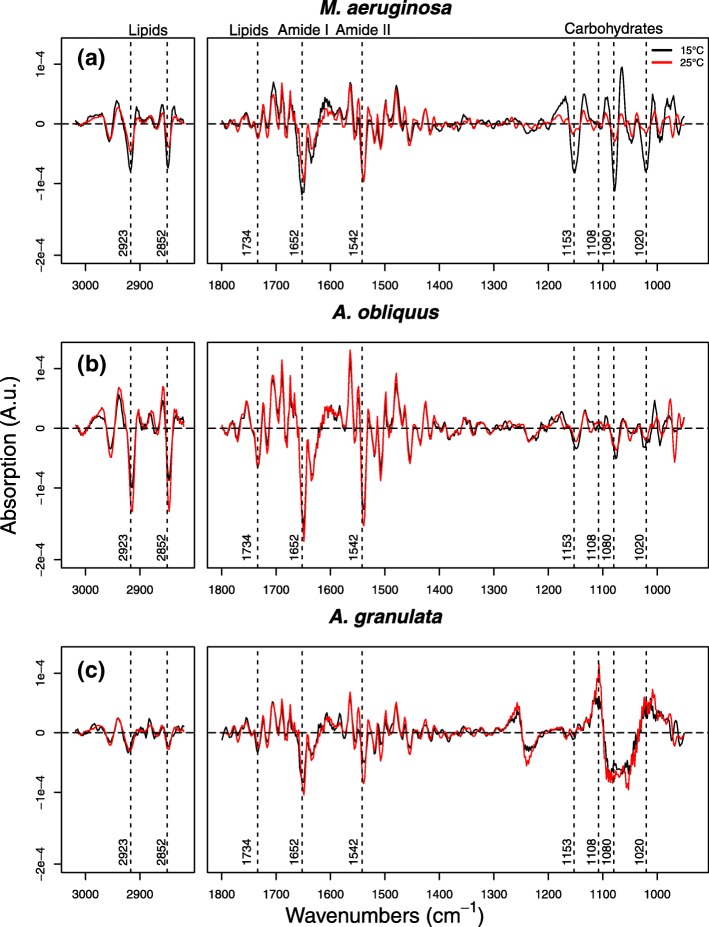
Average 2nd derivative spectra of *M. aeruginosa* (**a**), A. oliquus (**b**) and *A. granulata* (**c**) grown in the mock mixed community at 15 °C and 25 °C obtained by Synchrotron FTIR micro-spectroscopy. The vertical dashed lines identify the most biologically relevant bands of cell spectra, the corresponding wavenumbers are labelled. It must be kept in mind that different macromolecules may have overlapping absorption bands. For instance, although we refer to carbohydrates when considering the spectral window 950-1200 cm^-1^, other important macromolecules, such as nucleic acids, may contribute to spectral features present in this region

Average spectra of the phytoplankton species grown in a mixed assemblage at 15 °C and 25 °C obtained by Synchrotron FTIR micro-spectroscopy provide useful information on the biochemical composition of each population, which is typically consistent with the results of the biochemical assays. To highlight subtle changes associated to growth temperatures, average 2nd derivative spectra are shown in Fig. 3. *M. aeruginosa* presented stronger intensities of the bands found in the carbohydrate region at 15 °C, but just slightly different protein bands at the two temperatures. The spectra of *A. obliquus* were essentially the same, except for small differences present at the carbohydrate frequencies, where few peaks (1153 and 1020 cm^− 1^) were higher at 15 °C, than at 25 °C. Similarly, in *A. granulata*, the cell spectra differ mostly because of differences present in the spectral window between 950 and 1250 cm^− 1^, which is characteristic for carbohydrates and silica absorption (Fig. 3).

A PCA was performed on each species separately to explore how the populations differ biochemically at the two temperatures. In this case, the analysis is performed on the whole cell population and each spectrum corresponds to the overall composition of one single cell.

**Fig. 4:**
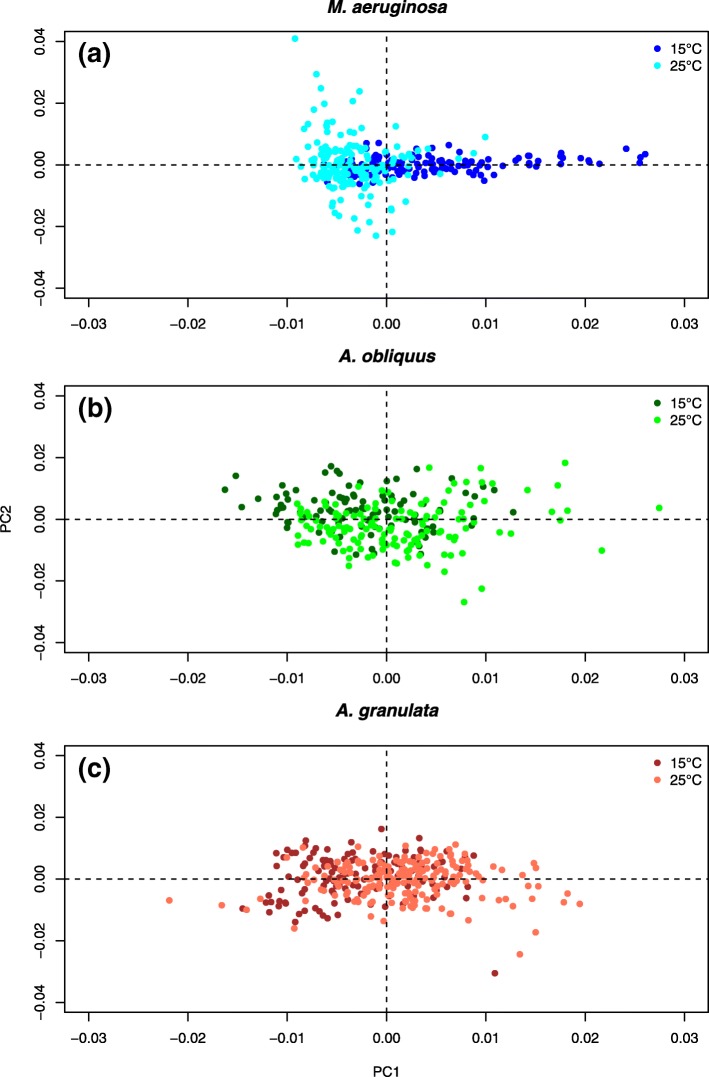
Scores plot of the PCA performed on single-cell FTIR spectra of *M. aeruginosa* (**a**), *A. oliquus* (**b**) and *A. granulata* (**c**) grown in the mixed assemblage at 15 °C and 25 °C

None of the considered populations showed a clear separation in the scores plot. Instead, a progressive trend could be recognized for all of them. This was more evident for the populations of *M. aeruginosa*, where the effect of the higher growth temperature was reflected in a clustering of cells at negative scores along PC1. Additionally, the loadings indicated that carbohydrates are the responsible compounds for this trend (Fig. 4, Additional file 5: Figure S2).

The trend is less evident but still detectable for *A. obliquus* and *A. granulata*. Here, a continuous gradient form negative to positive PC1 scores as a function of the growth temperature was observed. The loadings indicated that for both species, the protein bands were responsible for the separations of the cells along the PC1 (Additional file 5: Figure S2).

### Kernel density estimates for macromolecule-frequency distribution

Once calibrated and validated, the PLSr models were used to estimate the biochemical composition of single phytoplankton cell grown in a mock community. The frequency distributions of the results obtained by the predictive models were then studied by means of Kernel density estimates to characterize the heterogeneity of the community in macromolecular terms.

**Fig. 5:**
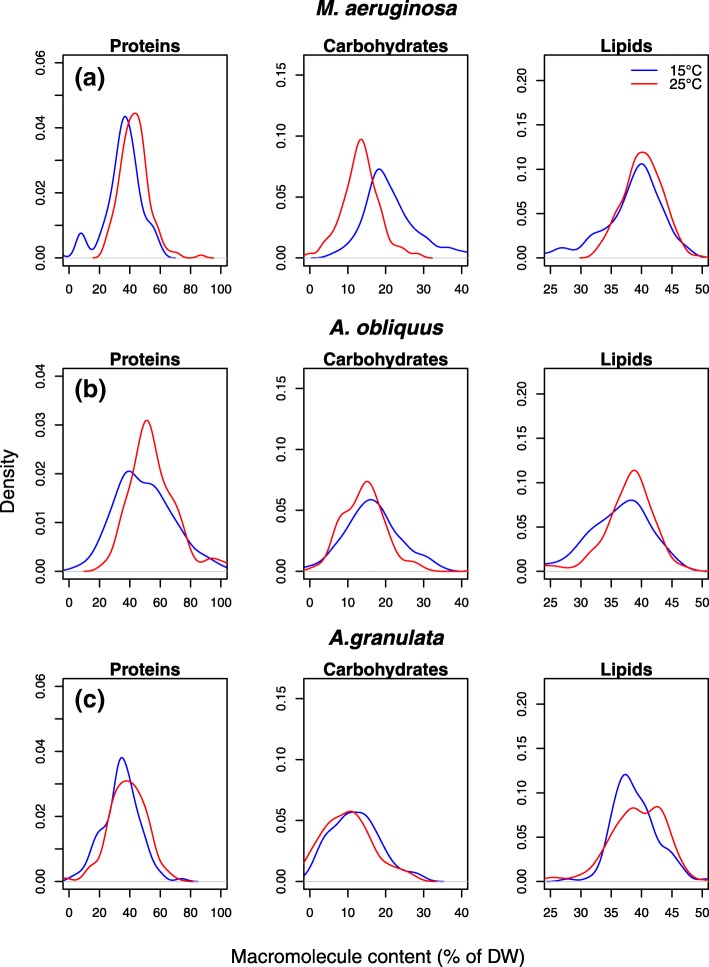
Kernel density distribution for the macromolecule contents of *M. aeruginosa* (**a**), *A. oliquus* (**b**) and *A. granulata* (**c**) grown in the mixed assemblage at 15 °C and 25 °C. The probability distributions are reported for the single species and for the whole community

The distribution of macromolecules was typically symmetric uni-modal, except for the protein content in *A. obliquus* at 15 °C and 25 °C, the protein content of *M. aeruginosa* at 25 °C and its carbohydrate content at 15 °C, where a secondary mode was observed (Fig. 5).

For each macromolecule, the mode of the predicted values resembled the quantification performed by reference methods, but it also emerged that the density profiles where mostly overlapping at the two temperatures (Fig. 5). Consistent with the results obtained from biochemistry and FTIR-spectroscopy, *M. aeruginosa* underwent the greatest changes in composition (i.e. the largest shift and separation of the density distributions), compared to all other species. The protein density distribution shifted towards higher values when the cells were grown at 25 °C. The opposite trend was observed for the carbohydrate content. The lipid distributions overlapped at the two temperatures (Fig. 5).

*A. obliquus* presented density distribution changes as a function of temperature resembling those of *M. aeruginosa*. At 15 °C, the distribution of proteins was bimodal, with the two modes being similar in intensity (Fig. 5). At 25 °C, the main mode was shifted towards higher protein contents the second mode was still present, but decreased in intensity. The mode of the carbohydrates was slightly higher at 15 °C, with respect to that of the cells grown at 25 °C, although a great proportion of cells exhibited similar carbohydrate contents (Fig. 5). Furthermore, at 25 °C the distribution was bimodal. The main mode of lipids was the same at 15 and 25 °C, but at 15 °C a second, less intense mode was also present.

*A. granulata* presented less prominent adjustments at the two temperatures (Fig. 5). Interesting, although protein and carbohydrate density distributions were mostly overlapping, the lipid one seemed to be more affected. At 25 °C, the distribution was bimodal and both modes were higher, with respect to the main one present at 15 °C.

## Discussion

### Macromolecular composition modeling

The developed PLSr models presented very high precision in the estimation of new samples (±2–6% of the predicted value) (Fig. 1, Table 2), reflecting the fact that FTIR-spectra contain major information regarding the macromolecular composition of phytoplankton [17].

From the analysis of the regression coefficients, it emerged that the prediction of each macromolecule was not exclusively dependent on spectral features related to that specific compound (e.g. the Amide I and II for proteins). Instead, the prediction of each compound was based on spectral bands corresponding to all major macromolecular pools present in the cells (Fig. 2). In our experiments, the prediction of protein, lipid and carbohydrate content relied on variations of the Amide I and II, on the C-O-C bonds of carbohydrates, as well as on the CH_2_ bonds in the acyl-chains of lipids (Fig. 2). This multiple-band dependency of macromolecule prediction is explained by the fact that an FTIR-spectrum represents a snapshot of the overall C-metabolism of a cell [23]. External perturbations typically induce a reallocation of C among all the major organic pools [3, 4, 29, 35]. For instance, under N-limitation a decrease in the protein content is generally accompanied by an increase of carbohydrates and/or lipids [3, 4]. It follows that the prediction of proteins may benefit, besides changes in the Amide I and II bands, also from changes in the spectral bands corresponding to lipids or carbohydrates. A similar finding was reported by Sackett et al. [23], which used spectral windows corresponding to lipids (CH_2_ stretching) and carbohydrates to predict the protein content in an Antarctic diatom. Finally, macromolecules present different vibrational extinction coefficients [15]. Therefore, the prediction of lipids, for example, is influenced more by changes in the protein content, because the Amides I and II present higher extinction coefficient with respect to the C=O bonds of lipids. Even if subjected to similar quantitative changes, those compounds will exhibit non-comparable changes in peak intensity.

### Single-cell analysis and population trait dynamics

Although Dean et al. [9, 26, 35] and Sackett et al. [14] used FTIR-spectroscopy on field samples, predictive models created in the laboratory have not been applied to natural samples yet. Here, a mock phytoplankton community was utilized to simulate the complexity of natural samples and to test for the first time the efficiency of the approach under controlled conditions. Once calibrated and validated, the PLSr models were used to estimate the absolute macromolecule contents in single phytoplankton cell scanned by Synchrotron FTIR micro-spectroscopy. The frequency distribution of the results obtained using the predictive models was then studied by means of Kernel density estimates to characterize the heterogeneity of the community in macromolecular terms.

From a quantitative point of view, the mode of each distribution resembles the changes identified by biochemistry and was in accordance with recent studies investigating the biochemical responses of phytoplankton to temperature (Fig. 5). The protein content appeared to be positively correlated to temperature, whereas storage and structural compounds, such as carbohydrates, had an opposite behavior [7, 25, 28, 36].

Interestingly, interspecific differences, in terms of macromolecular plasticity, emerged as a function of temperature (Fig. 5). *M. aeruginosa* and *A. obliquus* were the most plastic species whereas *A. granulata* underwent only minor changes in the distribution of macromolecules. As reported by Sackett et al. [14] this taxon-specific macromolecular plasticity is likely the consequence of different ecological niches occupied by the species. In support to this observation, Fanesi et al. [32] found for the same species comparable results about the photo-biology acclimation strategies in response to temperature.

A different picture emerged when considering the intra-population variability. As we hypothesized, the Kernel density estimations revealed that each phytoplankton population did not elect a uniform response with respect to temperature (Fig. 5). Similar results have been also reported earlier [10, 26], though the analysis of these authors refer to relative amount of macromolecules and not to absolute quantification. At the population level, the response seemed to be rather complex, ranging from curve shifts, to changes in skewedness and number of modes. Only a small proportion of cells presented temperature-specific phenotypes. Most of the remaining cells exhibited instead a pool of shared phenotypes at 15 °C and 25 °C. Therefore, the results from an averaged population could lead to erroneous interpretations, or, they will fail to represent the whole population dynamic with respect to an external cue [37, 38].

With the evolving of single-cell techniques, population heterogeneity has been interpreted as a functional trait, more than only as “black noise” resulting from stochastic events at the gene expression and translation levels [39–41]. For instance, there is evidence supporting the “bet hedging” theory: the coexistence of multiple phenotypes in a population, but only one suited for a specific set of conditions. Such a strategy confers resilience to external perturbations and/or access to multiple niches, rather than being just a passive consequence of biology nonlinearities [38]. This can be particularly advantageous in those environments characterized by rapid fluctuations of the external conditions (such as freshwater ecosystems), where a multitude of cell phenotypes may ensure a continuum of niches that can be occupied by a population. In these terms, the phenotypic heterogeneity of a population might be seen as a strategy, beside acclimation [41] and homeostasis [35, 42], aimed at maximizing species fitness with respect to the external *milieu*. We cannot exclude that the differences in growth and metabolic rates at the two temperatures (see Additional file 6: Table S1) might be responsible for some of the patterns identified by the single-cell analysis. For instance, at 25 °C the high synthetic rates for proteins [36] could in turn be affected by expression and translation errors, increasing the probability of generating and accumulating different phenotypes over-time.

Although our observations are based on microcosms and have to be further tested in natural habitats, they already provide important implications on what we know about communities modeling based on functional traits [43–45]. For example, the identification of multiple phenotypes (and therefore of multiple functional units) in a clonal population may make it more difficult to include a specie in a defined functional group [6]. It is evident that what bulk analysis reflects just the tip of the iceberg hiding complex population dynamics. More research will be necessary to understand to which extent cell functionality is overlapping with the heterogeneity of a population and to deeply understand the role played in the response of phytoplankton populations to external perturbations.

## Conclusions

In this study we used the PLSr algorithm to correlate FTIR-spectra to the main macromolecules that make-up phytoplankton cells (i.e. proteins, lipids and carbohydrates). The models were used to predict the macromolecular content of single-cells (scanned by Synchrotron micro FTIR spectroscopy), in a mock phytoplankton assemblage to study the effect of temperature on C-allocation. The results showed that a great proportion of cells was expressing a chemical phenotype specifically acclimated to the growth temperature. At the same time, another multitude of “non-acclimated” phenotypes were present in the same population. This wide population heterogeneity may be of advantage in response to rapid and drastic changes of the external conditions. Indeed, the presence of cells with a chemical phenotype that fits already with new physical settings can allow a fast response avoiding the acclimation steps of pre-existing cells.

From a technical point of view, this approach may find application in ecological studies of phytoplankton dynamics. Indeed, a single spectral dataset may be calibrated to predict several different phenotypic traits (e.g. macromolecules, elemental ratios, growth rate etc.), opening the opportunity to obtain a complete “check-up” of single-cells in field samples and better resolve biomass quality and production in aquatic ecosystems.

## Methods

### Culture maintenance

The species used in this study were selected as a function of their ecological importance and ability to grow under laboratory conditions. Stock cultures of *Microcystis aeruginosa*, *Acutodesmus obliquus* and *Aulacoseira granulata* were grown semi-continuously in 1 L Erlenmeyer flasks filled with 500 mL of WC growth medium [46] and maintained at 15 and 25 °C at a photon flux density of 140 μmol photons m^− 2^ s^− 1^ in a light:dark cycle of 14:10 h. The WC medium was modified respect to the original recipe by adding the vitamin solution of the Diatom medium [47] and double amount of silica.

To simulate the conditions of a natural-like phytoplankton community, the stock mono cultures of the three species were diluted to a Chl*a* concentration of 0.01 mg L^− 1^, and then mixed together in equal amounts to a final volume of 500 mL in 1 L Erlenmeyer flasks to create a mock community. For all the experiments, the cultures were grown in batch for no longer than 1 week and the cells were harvested in the early-mid exponential phase (typically after 5–6 days from the initial inoculum). Growth was followed by daily microscopic cell counts of each species both in the monoculture and in the mixed communities. Growth rate was determined as the slope of a linear regression of the natural logarithm of cell counts against time (Additional file 6: Table S1).

### Biochemical characterization of phytoplankton cells by “wet methods”

The biochemical quantification of macromolecules was performed on pure cultures of each single species and for each growth condition. The assays were repeated on 5 independent biological replicates (i.e. on 5 distinct cultures). Statistically different means were identified by t-test with a significance level set at 95%.

#### Dry weight quantification

For the determination of cell dry weight, 400 mL of cell suspension were centrifuged at 2000×g for 10 min at 20 °C. The pellet was washed in 2 mL of distilled water and centrifuged in pre-weighted Eppendorf tubes (mini-spin, Eppendorf, Germany) at 5000×g for 10 min at room temperature. Cell pellet was then frozen in liquid nitrogen and freeze-dried overnight. The Eppendorf tubes containing the dry cell pellet were then weighted in an analytical balance for the estimation of the dry weight.

#### Protein quantification

30 mL of cell suspension (approximately 0.5–1 mg DW) were centrifuged at 2000×g for 10 min at 20 °C. The pellet was washed in 2 mL of distilled water and centrifuged (mini-spin, Eppendorf, Germany) at 5000×g for 10 min at room temperature. Cells were finally frozen in liquid nitrogen and subsequently freeze-dried overnight and stored in closed Eppendorf tubes inside a desiccator until the measurements were performed.

The samples were resuspended in 1 mL HCl (18%; 6 N) in 2 mL Eppendorf tubes and hydrolyzed in a thermo-shaker at 100 °C, 800 rpm for 24 h. After centrifugation (12,000×g for 10 min at room temperature), the supernatant was transferred to another Eppendorf tube. Aliquots of 100 μL were transferred to fresh tubes and the acid evaporated in a vacuum concentrator for 1.5 h. Protein quantification was performed according to the Ninhydrin-based assay of Starcher [48] using bovine serum albumin (Carl Roth GmbH, Germany) as a calibration standard. Absorption was measured spectrophotometrically at 575 nm (Specord 250, Analytic Jena, Germany).

#### Carbohydrate quantification

30 mL of cell suspension were harvested as described above. Carbohydrates were quantified according to the phenol-sulfuric acid method described in Dubois et al. [49]. The samples were resuspended in 1 mL of H_2_SO_4_ (98%; *v*/v) in 2 mL Eppendorf tubes and hydrolyzed in a thermo-shaker at 80 °C, 800 rpm for 2 h. Aliquots (10 μL or 100 μL) of the samples were then transferred to new Eppendorf tubes and diluted (1:10 or 1:100, final volume = 1 mL) depending on the initial cells density. Finally, 100 μL of phenol (0.1%; *w*/*v*) were added to the samples and cooled down at room temperature. Absorption was measured spectrophotometrically at 485 nm (Specord 250, Analytic Jena, Germany) using glucose as a calibration standard.

#### Lipid quantification

The lipid quantification was carried out gravimetrically according to Lee et al. [50]. Cells were harvested as described above but starting with 400 mL of cultures (approximately 7–10 mg DW). The freeze-dried pellet was first resuspended in 2 mL of phosphate buffer (0.1 M and pH 7.4) to avoid side-reactions. Samples were than cooled down in ice and then disrupted with glass beads in a cell homogenizer for 40 s at 6000 rpm (Precellys 24, Bertin Technologies, Erlangen, Germany). The process was repeated 3 times. The suspension was then cooled and maintained in ice in the dark to avoid photo-oxidation. The extract was transferred into sealed glass tubes and organic solvent (approx. 4–5 mL of a mixture of chloroform:methanol 2:1 *v*/v) was added to create a phase separation. The mixture was mixed vigorously and centrifuged at 2400×g for 10 min to obtain a clean phase separation. After the centrifugation step, the lower phase (containing the lipids extract) was removed with a Hamilton syringe, transferred into sealed glass tubes and maintained in the dark and in ice. The extraction step was repeated (typically two times) until the cell pellet interposed between the organic solvent and the water phase resulted completely bleached.

Finally, the organic solvent was evaporated to dryness in a chemical cabinet under a stream of N_2_ and the lipids quantified gravimetrically in an analytical balance.

#### Biogenic silica (frustules of *A. granulata*) quantification

For the quantification of silica (Si), a protocol based on a gravimetric quantification was developed. 50–70 mL of culture suspension (approximately 1.5–2 mg DW) was harvested as described above. The pellet was resuspended in 1 mL of distilled water and further diluted with NaClO (12% v/v) to a final concentration of 6% (v/v) in 2 mL final volume. The suspension was then mixed and maintained at room temperature in the dark for no more than 1.5 h. During this step, all the organic compounds are detached from frustules. The frustules were separated from the organic suspension by a short centrifugation spin, the supernatant discharged, and the pellet resuspended and washed in distilled water 5–6 times to get rid of the residual organic material and NaClO. A further washing step was performed in 100% Acetone (v/v) and finally the sample was washed in distilled water, centrifuged and the frustule pellet dried in an oven at 40 °C.

### Biochemical characterization of phytoplankton cells by FTIR-spectroscopy

#### Bench-top FTIR-spectroscopy measurements: sample preparation and spectra acquisition

In order to generate predictive models for the macromolecule quantification, the same samples that were assayed by “wet” biochemical methods were also measured using a bench-top FTIR-spectrometer. Bench-top FTIR measurements have been performed as described in detail elsewhere [28]. Briefly, 1.5–2 mL of cell suspension were harvested by gentle filtration, resuspended and washed in distilled water to remove salts and cell debris. The sample were then centrifuged (8000 g for 8 min.) and resuspended in 10–20 μL of distilled water. Finally, 2 μL of the algal suspension were deposited on a silicon micro plate (384 wells, Bruker) and dried in a cabinet drier. Spectra were recorded in transmission mode with 32 scans co-added and averaged in the spectral range 4000–700 cm^− 1^ with a resolution of 4 cm^− 1^. Background signal was measured scanning an empty well using the same acquisition settings.

#### Chemometric analysis of FTIR-spectra: the PLSr predictive models

The quantitative relationship between FTIR-spectra and macromolecule contents, as determined by reference methods (i.e. the biochemical assays), was established calibrating three PLSr models [31], one for each macromolecular pool (i.e. proteins, lipids and carbohydrates). Each PLSr model was calibrated using the spectra obtained from FTIR bench-top measurements of three phytoplankton pure cultures grown at 15 °C and 25 °C as the predictor matrix, and the respective biochemistry data as the response one [28, 29]. The development of models based on different taxa was preferred to the use of species-specific ones because they would incorporate different C partitioning strategies resulting from the evolutionary trajectory of phytoplankton taxa [4].

Spectra pre-processing was performed as described in detail in Fanesi et al. [28]. Briefly, the spectra were exported from the OPUS software (v.5.0; Bruker Optics, Ettlingen, Germany) onto the R environment (version 3.2.3) [51], cut in the spectral ranges 3019–2819 and 1800–950 cm^− 1^, converted to 2nd derivatives by the Savitzky-Golay algorithm [52] using a quadratic polynomial function with nine smoothing points and finally mean centered.

The PLSr has been implemented in the R software using the “pls” package developed by Mevik and Wehrens [53]. The orthogonal scores algorithm was used for the PLSr. The model fit quality was studied calculating the R^2^ and the root mean squared error of calibration (RMSEC) of each macromolecular pool. The prediction performance of the model was inferred from the root mean squared error of prediction (RMSEP, expressed in % of DW) calculated from the leave-one-out cross-validation (LOOCV). The regression coefficients were analyzed to relate spectral features to the macromolecule content in the cells.

### Application of the PLSr predictive models for single-cell analysis

#### Synchrotron FTIR micro-spectroscopy measurements: sample preparation and spectra acquisition

Once calibrated and validated, the PLSr predictive models were applied to a mock phytoplankton community grown at two temperatures (15 °C and 25 °C) to study the effect of temperature on the macromolecular content of single cells. However, since standard bench-top FTIR-spectrometers do not have the resolution required for single-cell measurements, the single phytoplankton cell spectra were acquired by means of Synchrotron FTIR micro-spectroscopy at the Italian Synchrotron in Trieste (Elettra-Sincrotrone Trieste, proposal number 20145158, Beamline SISSI, Chemical and Life Sciences branch, Basovizza, Trieste, Italy) [54].

Aliquots (2 mL) of the mock community were centrifuged and then fixed in a phosphate buffered saline solution (2 mL of PBS; 0.1 M, pH 7.4) and 2% (*v*/v) formalin and maintained in the dark at 4 °C until the measurement were performed (less than 2 month). Before the measurements, all samples were washed twice in distilled water to remove PBS residuals and formalin from the cells. The cells were finally resuspended in 100 μL of distilled water; an aliquot of 2 μL was deposited on a CaF_2_/Si sample holder and dried at room temperature under a sterile bench.

Spectra were recorded with a Hyperion 3000 IR microscope coupled to a Bruker Vertex 70 interferometer (Bruker Optics, Ettlingen, Germany) connected to the synchrotron IR beamline. Spectra were acquired in transmission mode with 256 scans co-added and averaged in the spectral range 4000–700 cm^− 1^ with a spectral resolution of 4 cm^− 1^ and a lateral resolution set according to the cell dimension (from 8 × 8 μm to 10 × 10 μm). The measurement of an empty spot was used as a background, collected with the same instrumental settings. Mixed community experiments were repeated in duplicate for each growth condition (i.e. two distinct cultures). At least 50 cells per sample (i.e. each species at each condition) were measured, resulting in a total of 998 spectra for the whole mock community dataset.

Spectra preprocessing for the mixed community dataset was performed as described above. The macromolecular composition of the single cells was then estimated based on the PLSr models using the function “prediction” present in the “pls” package. In this way for each single-cell spectrum obtained at the Synchrotron, the protein, carbohydrate and lipid contents could be assessed.

The single-cell spectra of each species present in the mixed assemblage were further analyzed by PCA (package “vegan”) [55] to explore the dataset and highlight qualitative differences as a function of temperature.

#### Kernel density estimator for the mock community analysis

The Kernel density estimator (KDE) [56, 57] was applied on the values (i.e. the single-cell protein, carbohydrate and lipid contents) predicted by the PLSr models to study the macromolecule-frequency distribution as a function of temperature in the mock community. The KDE has been used because it confers more resolved frequency distributions in comparison to standard methods, such as histograms. The KDE was computed using the basic statistic package present in the R software setting the standard deviation of the smoothing kernel (Gaussian) as the smoothing bandwidth.

## Additional files

Additional file 1: **Figure S1.** Prediction plots of protein, carbohydrate and lipid content. (PDF 159 kb)

Additional file 2: **Table S2.** Percentage of explained variance from the PLSr models for protein prediction. (PDF 7 kb)

Additional file 3: **Table S3.** Percentage of explained variance from the PLSr models for carbohydrate prediction. (PDF 7 kb)

Additional file 4: **Table S4.** Percentage of explained variance from the PLSr models for lipid prediction. (PDF 7 kb)

Additional file 5: **Figure S2.** Loadings plot of the PCA performed on single-cell FTIR microspectra. (PDF 237 kb)

Additional file 6: **Table S1.** Growth rate μ[d^-1^] in response to two different growth temperatures of the diatom *Aulacoseira granulata,* the green alge *Acutodesmus obliquus* and the cyanobacteria *Microcystis aeruginosa* grown as uni-algal cultures or together in a mock community. (PDF 179 kb)

## Abbreviations

C: Carbon
Chla: Chlorophyll a
DW: Dry weight
FTIR: Fourier Transform Infrared
KDE: Kernel density estimator
LOOCV: Leave-one-out cross-validation
PBS: Phosphate buffered saline solution
PC: Principal components
PCA: Principal component analysis
PLSr: Partial least square regression
RMSEC: Root mean squared error of calibration
RMSEP: Root mean squared error of prediction
Si: Silica
