# Supplementary material for: Quantitative macromolecular patterns in phytoplankton communities resolved at the taxonomical level by single-cell Synchrotron FTIR-spectroscopy
Source: BMC Plant Biol. 2019 Apr 15;19:142. doi: 10.1186/s12870-019-1736-8 (PMC6466684; doi:10.1186/s12870-019-1736-8)
Supplement: Supplementary file 1 — Figure S1. Prediction plots of protein, carbohydrate and lipid content. (PDF 159 kb) [file 12870_2019_1736_MOESM1_ESM.pdf]

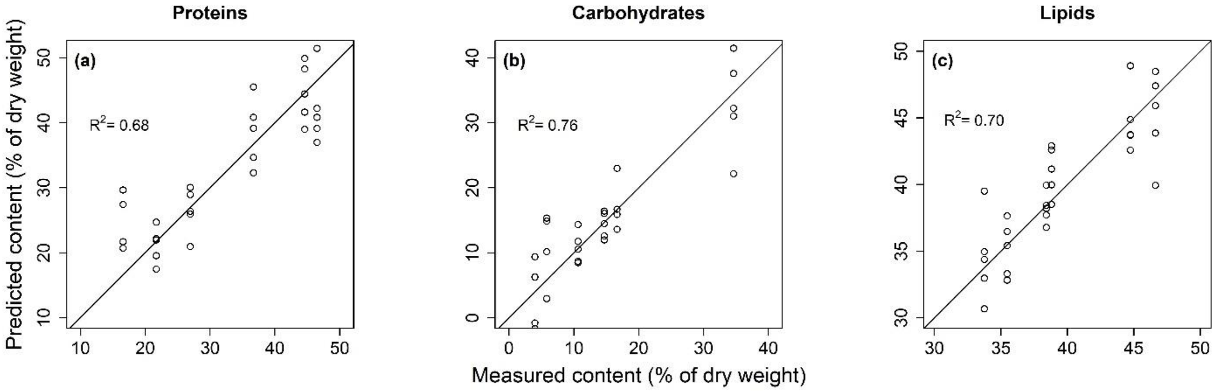

Figure S1: Prediction plots of protein, carbohydrate and lipid content. Prediction estimates were obtained using the Leave One Out Cross Validation (LOOCV) on the PLSr models calibrated with bench-top FTIR-spectra and "wet" biochemical assays as a reference method. Each model was calibrated using three phytoplankton species (a diatom, a cyanobacterium and a green alga) grown at two temperatures (15 and 25°C). The macromolecule content is expressed as % of dry weight and the R^2 for each regression is also reported. The number of PLS-PCs used was 7.
