# Supplementary material for: Quantitative macromolecular patterns in phytoplankton communities resolved at the taxonomical level by single-cell Synchrotron FTIR-spectroscopy
Source: BMC Plant Biol. 2019 Apr 15;19:142. doi: 10.1186/s12870-019-1736-8 (PMC6466684; doi:10.1186/s12870-019-1736-8)
Supplement: Supplementary file 2 — Table S2. Percentage of explained variance from the PLSr models for protein prediction. (PDF 7 kb) [file 12870_2019_1736_MOESM2_ESM.pdf]

**Table S2:** Percentage of explained variance in the predictor and response matrix obtained from the PLSr models calibrated for the prediction of phytoplankton proteins.

| | PLS-PLC1 | PLS-PLC2 | PLS-PLC3 | PLS-PLC4 | PLS-PLC5 | PLS-PLC6 | PLS-PLC7 |
|---|---|---|---|---|---|---|---|
| Predictor matrix | 82.55 | 94.26 | 97.04 | 98.44 | 98.85 | 99.09 | 99.38 |
| response | 54.08 | 67.82 | 76.20 | 82.68 | 88.54 | 93.07 | 94.65 |
