# Supplementary material for: Quantitative macromolecular patterns in phytoplankton communities resolved at the taxonomical level by single-cell Synchrotron FTIR-spectroscopy
Source: BMC Plant Biol. 2019 Apr 15;19:142. doi: 10.1186/s12870-019-1736-8 (PMC6466684; doi:10.1186/s12870-019-1736-8)
Supplement: Supplementary file 3 — Table S3. Percentage of explained variance from the PLSr models for carbohydrate prediction. (PDF 7 kb) [file 12870_2019_1736_MOESM3_ESM.pdf]

Table S3: Percentage of explained variance in the predictor and response matrix obtained from the PLSr models calibrated for the prediction of phytoplankton carbohydrates.

	PLS-PLC1	PLS-PLC2	PLS-PLC3	PLS-PLC4	PLS-PLC5	PLS-PLC6	PLS-PLC7
Predictor matrix	82.75	89.22	97.54	98.47	98.93	99.15	99.38
response	42.28	75.99	85.32	90.89	92.66	94.50	96.03
