# Supplementary material for: Quantitative macromolecular patterns in phytoplankton communities resolved at the taxonomical level by single-cell Synchrotron FTIR-spectroscopy
Source: BMC Plant Biol. 2019 Apr 15;19:142. doi: 10.1186/s12870-019-1736-8 (PMC6466684; doi:10.1186/s12870-019-1736-8)
Supplement: Supplementary file 4 — Table S4. Percentage of explained variance from the PLSr models for lipid prediction. (PDF 7 kb) [file 12870_2019_1736_MOESM4_ESM.pdf]

Table S4: Percentage of explained variance in the predictor and response matrix obtained from the PLSr models calibrated for the prediction of phytoplankton lipids.

	PLS-PLC1	PLS-PLC2	PLS-PLC3	PLS-PLC4	PLS-PLC5	PLS-PLC6	PLS-PLC7
Predictor matrix	82.32	94.41	96.60	98.47	98.84	99.07	99.37
response	50.08	67	75.17	79.46	85	89	91.54
