# Supplementary material for: Quantitative macromolecular patterns in phytoplankton communities resolved at the taxonomical level by single-cell Synchrotron FTIR-spectroscopy
Source: BMC Plant Biol. 2019 Apr 15;19:142. doi: 10.1186/s12870-019-1736-8 (PMC6466684; doi:10.1186/s12870-019-1736-8)
Supplement: Supplementary file 5 — Figure S2. Loadings plot of the PCA performed on single-cell FTIR microspectra. (PDF 237 kb) [file 12870_2019_1736_MOESM5_ESM.pdf]

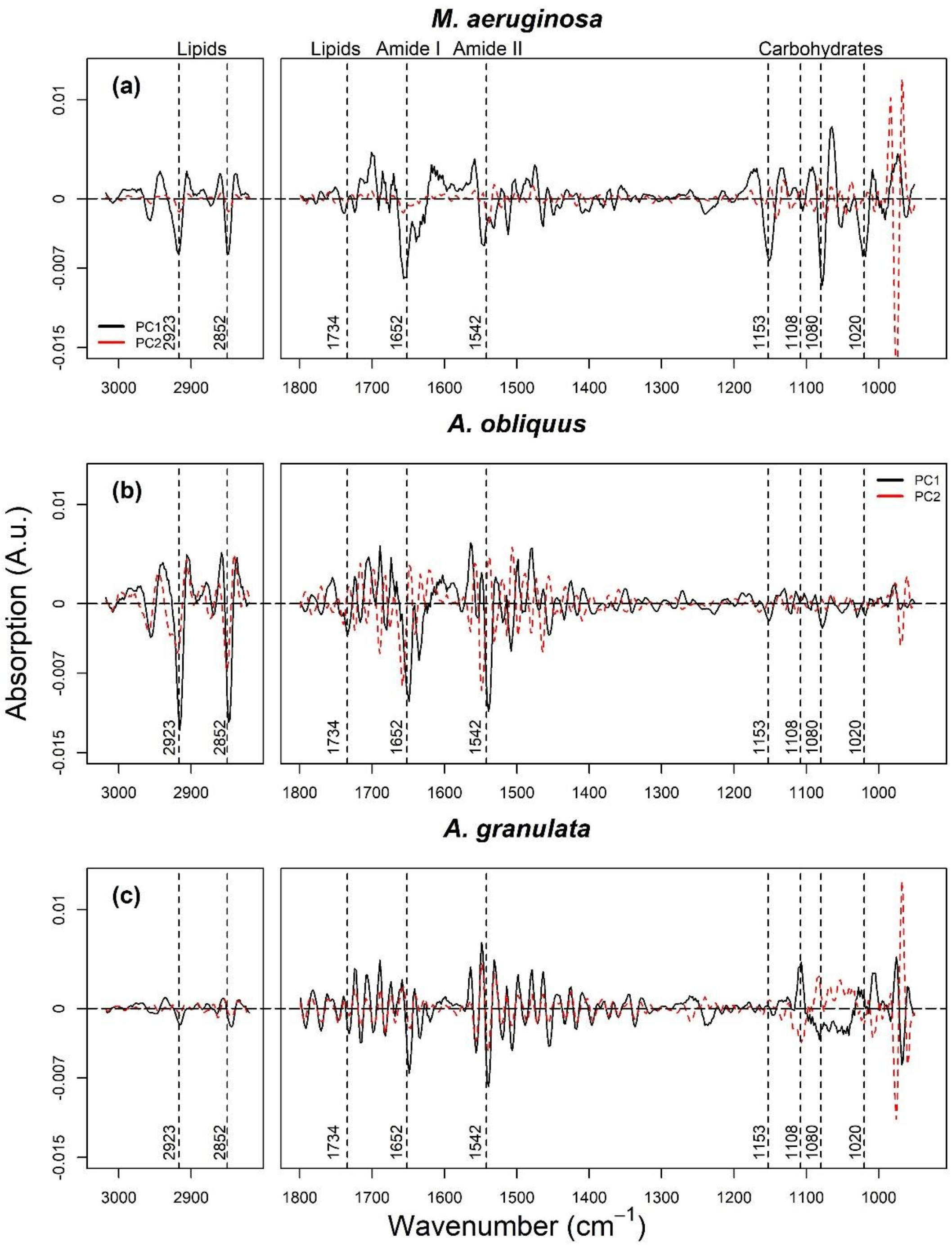

Figure S2: Loadings plot of the PCA performed on single-cell FTIR microspectra of *M. aeruginosa* (a), *A. oliquus* (b) and *A. granulata* (c) grown in the mixed assemblage at 15 and 25°C. The vertical dashed lines identify the most biologically relevant bands of cell spectra, the corresponding wavenumbers are labelled.
