# Supplementary material for: Quantitative macromolecular patterns in phytoplankton communities resolved at the taxonomical level by single-cell Synchrotron FTIR-spectroscopy
Source: BMC Plant Biol. 2019 Apr 15;19:142. doi: 10.1186/s12870-019-1736-8 (PMC6466684; doi:10.1186/s12870-019-1736-8)
Supplement: Supplementary file 6 — Table S1. Growth rate μ[d-1] in response to two different growth temperatures of the diatom Aulacoseira granulata, the green alge Acutodesmus obliquus and the cyanobacteria Microcystis aeruginosa grown as uni-algal cultures or together in a mock community. (PDF 179 kb) [file 12870_2019_1736_MOESM6_ESM.pdf]

Table S1: Growth rate μ[d^{-1}] in response to two different growth temperatures of the diatom *Aulacoseira granulata,* the green alge *Acutodesmus obliquus* and the cyanobacteria *Microcystis aeruginosa* grown as uni-algal cultures or together in a mock community.

	A. granulata		*A. obliquus*		*M. aeruginosa*	
	15 °C	25 °C	15 °C	25 °C	15 °C	25 °C
Monoculture	0.66 (0.19)	0.8 (0.21)	0.53 (0.14)	0.87 (0.08)	0.23 (0.03)	0.74 (0.12)
Mock community	0.83 (0.12)	0.92 (0.06)	0.76 (0.03)	1.06 (0.05)	0.24 (0.01)	0.82 (0.03)
